# Primary Tumor Sidedness, RAS and BRAF Mutations and MSI Status as Prognostic Factors in Patients with Colorectal Liver Metastases Treated with Surgery and Thermal Ablation: Results from the Amsterdam Colorectal Liver Met Registry (AmCORE)

**DOI:** 10.3390/biomedicines9080962

**Published:** 2021-08-05

**Authors:** Madelon Dijkstra, Sanne Nieuwenhuizen, Robbert S. Puijk, Florentine E. F. Timmer, Bart Geboers, Evelien A. C. Schouten, Jip Opperman, Hester J. Scheffer, Jan J. J. de Vries, Kathelijn S. Versteeg, Birgit I. Lissenberg-Witte, M. Petrousjka van den Tol, Martijn R. Meijerink

**Affiliations:** 1 Department of Radiology and Nuclear Medicine, Amsterdam University Medical Centers, VU Medical Center Amsterdam, Cancer Center Amsterdam, 1081 HV Amsterdam, The Netherlands; s.nieuwenhuizen1@amsterdamumc.nl (S.N.); r.puijk@amsterdamumc.nl (R.S.P.); f.timmer1@amsterdamumc.nl (F.E.F.T.); b.geboers@amsterdamumc.nl (B.G.); e.schouten@amsterdamumc.nl (E.A.C.S.); hj.scheffer@amsterdamumc.nl (H.J.S.); j.devries1@amsterdamumc.nl (J.J.J.d.V.); mr.meijerink@amsterdamumc.nl (M.R.M.); 2 Department of Radiology and Nuclear Medicine, Noordwest Ziekenhuisgroep, Location Alkmaar, 1800 AM Alkmaar, The Netherlands; j.opperman@nwz.nl; 3 Department of Medical Oncology, Amsterdam University Medical Centers, VU Medical Center Amsterdam, Cancer Center Amsterdam, 1081 HV Amsterdam, The Netherlands; k.versteeg@amsterdamumc.nl; 4 Department of Epidemiology and Data Science, Amsterdam University Medical Centers, VU Medical Center Amsterdam, Vrije Universiteit Amsterdam, 1081 HV Amsterdam, The Netherlands; b.lissenberg@amsterdamumc.nl; 5 Department of Surgery, Amsterdam University Medical Centers, VU Medical Center Amsterdam, Cancer Center Amsterdam, 1081 HV Amsterdam, The Netherlands; mp.vandentol@amsterdamumc.nl

**Keywords:** colorectal cancer (CRC), colorectal liver metastases (CRLM), primary tumor sidedness, local tumor progression, surgery, thermal ablation, microwave ablation (MWA), radiofrequency ablation (RFA), RAS mutation, BRAF mutation, microsatellite instability (MSI)

## Abstract

The aim of this study was to assess primary tumor sidedness of colorectal cancer (CRC), rat sarcoma viral oncogene homolog (RAS) and v-raf murine sarcoma viral oncogene homolog B (BRAF) mutations and microsatellite instability (MSI) status as prognostic factors predicting complications, survival outcomes, and local tumor progression (LTP) following surgery and thermal ablation in patients with colorectal liver metastases (CRLM). This Amsterdam Colorectal Liver Met Registry (AmCORE) based study included 520 patients, 774 procedures, and 2101 tumors undergoing local treatment (resection and/or thermal ablation) from 2000 to 2021. Outcomes following local treatment were analyzed for primary tumor sidedness of CRC, RAS, and BRAF mutations and MSI status. The Kaplan–Meier method was used to estimate local tumor progression-free survival (LTPFS), local control (LC), distant progression-free survival (DPFS), and overall survival (OS). Uni- and multivariable analyses were performed based on Cox proportional hazards model. The chi-square test was used to analyze complications. Complications (*p* = 0.485), OS (*p* = 0.252), LTPFS (*p* = 0.939), and LC (*p* = 0.423) was not associated with tumor-sidedness. Compared to right-sided colon cancer (CC) (reference HR 1.000), DPFS was superior for left-sided CC and rectal cancer (*p* = 0.018) with an HR for left-sided CC of 0.742 (95% CI, 0.596–0.923) and for RC of 0.760 (95% CI, 0.597–0.966). Regarding RAS mutations, no significant difference was found in OS (*p* = 0.116). DPFS (*p* = 0.001), LTPFS (*p* = 0.039), and LC (*p* = 0.025) were significantly lower in the RAS mutation group. Though no difference in LTPFS was found between RAS wildtype and RAS mutated CRLM following resection (*p* = 0.532), LTPFS was worse for RAS mutated tumors compared to RAS wildtype following thermal ablation (*p* = 0.037). OS was significantly lower in the BRAF mutation group (*p* < 0.001) and in the MSI group (*p* < 0.001) following local treatment, while both did not affect DPFS, LTPFS, and LC. This AmCORE based study suggests the necessity of wider margins to reduce LTP rates in patients with RAS mutated CRLM, especially for thermal ablation. Upfront knowledge regarding molecular biomarkers may contribute to improved oncological outcomes.

## 1. Introduction

Colorectal cancer (CRC) represents approximately 10% of the annual global cancer incidence and is the second frequent cause of death from cancer worldwide [1]. The presence of distant metastases, mostly involving the liver, substantially affects the prognosis of patients with CRC. Colorectal liver metastases (CRLM) develop in up to 50% of all patients with CRC during the course of disease [2,3]. Five-year overall survival (OS) in untreated CRLM is <3% and when administering palliative chemotherapy about 11% [4,5,6,7]. Local treatment of CRLM with curative intent, comprising partial hepatectomy and thermal ablation (i.e., radiofrequency ablation (RFA), microwave ablation (MWA)), reaches 5-year OS of 50–60% [8,9,10,11,12,13].

According to the clinical risk score (CRS) for partial hepatectomy and the modified CRS for thermal ablation, well-known prognostic factors are size of the largest CRLM, number of CRLM and carcinoembryonic antigen (CEA) levels at the time of initial diagnosis of metastatic disease, node positive disease and a short (<12 months) disease-free interval between the primary tumor diagnosis and detection of CRLM [14,15,16,17].

There is growing attention for the prognostic role of the primary tumor location. Since the right colon (midgut), left colon and rectum (hindgut) develop from different embryological origins, tumor biology varies between right-sided colon cancer (CC), left-sided CC, and rectal cancer (RC) [18]. Primary tumors originating from right-sided CC are associated with a higher frequency of rat sarcoma viral oncogene homolog (RAS) mutations, v-raf murine sarcoma viral oncogene homolog B (BRAF) mutations, and microsatellite instability (MSI) [19,20]. Furthermore, right-sided CC showed a worse prognosis in both non-metastatic [21] and metastatic setting [22].

The impact of primary tumor location is relatively well established in outcomes following partial hepatectomy. Although Makowiec et al. found no significant difference in 5-year OS between patients with right-sided versus left-sided CRC (*p* = 0.64) [23], Dupré et al. demonstrated reduced OS rates for right-sided compared to left-sided CC (34.6 versus 45.3 months, *p* = 0.035) [24]. This was confirmed by Creasy et al. (hazard ratio (HR), 1.22; 95% CI, 1.02–1.45; *p* = 0.028) [25]. In addition, Zhang et al. observed an HR of 0.659 (95% CI, 0.478–0.910; *p* = 0.011) in recurrence-free survival comparing left-sided CC to right-sided CC after partial hepatectomy [26].

The influence of the primary tumor location on results following thermal ablation, are established by Gu et al. and Zhou et al. [27,28]. Following outcomes after RFA, patients with right-sided CC compared to left-sided CC had a median OS of 29.4 versus 40.3 months, respectively (*p* = 0.042) [27]. Moreover, comparison of right-sided CC versus left-sided CC showed a significant difference in progression-free survival (*p* = 0.012) in patients treated with MWA [28]. However, to our knowledge, additional research on the role of the primary tumor location in local tumor progression-free survival (LTPFS), distant progression-free survival (DPFS) and OS after thermal ablation for CRLM is limited.

This Amsterdam Colorectal Liver Met Registry (AmCORE) based study aimed to assess primary tumor sidedness of CRC, RAS and BRAF mutations and MSI status as prognostic factors predicting complications, survival outcomes and local tumor progression (LTP) following surgery and thermal ablation in patients with CRLM.

## 2. Materials and Methods

This single-center prospective cohort study was executed at the Amsterdam University Medical Centers—location VU University Medical Center, The Netherlands, a tertiary referral center for hepatobiliary and gastrointestinal malignancies. The AmCORE prospectively maintained CRLM database was used for extraction of data. The affiliated Institutional Review Board approved this study (METc Amsterdam University Medical Centers, location VUmc 2021.0121,). The ‘Strengthening the Reporting of Observational studies in Epidemiology’ (STROBE) guideline was used to report the analyzed study data [29].

### 2.1. Patient Selection

Data of all patients with CRLM were extracted from the prospective database. Additional recollecting of data was performed by retrospectively searching the hospital’s electronic patient database when required. Obtained data involved per-patient, per-procedure and per-tumor data. Patients undergoing surgical resection and thermal ablation were included. Patients undergoing stereotactic body radiation therapy (SBRT), irreversible electroporation (IRE), no, unknown, or planned local treatment, with loss to follow-up or unknown primary tumor location, were excluded.

### 2.2. Local Treatment

CRLMs were detected by cross-sectional imaging containing contrast enhanced computed tomography (ceCT) and 18F-fluoro-2-deoxy-d-glucose (^18^F-FDG) positron emission tomography (PET)—CT scans, using contrast enhanced magnetic resonance imaging (ceMRI) with diffusion-weighted images prior to local treatment. The multidisciplinary tumor board evaluations attended by (interventional) radiologists, oncological or hepatobiliary surgeons, medical oncologists, radiation oncologists, nuclear medicine physicians, gastroenterologists, and pathologists, determined the selection for local treatment and treatment strategy.

The surgical extent, specific technique, approach, and resection margins (with the intention and preoperative estimation of a possible pathological R0 resection) were at the discretion of the performing oncological or hepatobiliary surgeon. Metasectomy was preferred to preserve future liver volume when possible and anatomical resection when necessary.

Thermal ablation was performed by an experienced interventional radiologist (mastery degree in image-guided tumor ablation, having performed or supervised >100 thermal ablation procedures), preferably using real-time fluoroscopy computed tomography (CT)-guided (±ultrasound) percutaneous approach. Open approaches were performed when CRLM was potentially resectable, CRLMs > 3 and when distancing of certain delicate structures, such as intestines if a pneumo- or hydrodissection was unfeasible. Though not exclusively, RFA was mostly performed using the RF3000 generator with expandable LeVeen electrodes (Boston Scientific, Marlborough, MA, USA), the RITA system with compatible expandable electrodes (AngioDynamics BV, Amsterdam, The Netherlands) and MWA using the Evident or Emprint (Medtronic-Covidien, Minneapolis, MN, USA) or Solero (AngioDynamics BV, Amsterdam, The Netherlands) generators with compatible antennas. The ablations were performed conformal to the instructions for use provided by the manufacturer and in accordance with the CIRSE quality improvement guidelines (with an intentional tumor free ablation margin > 1 cm) [30]. Residual unablated tumor tissue in case of presumed insufficiently ablated margins were retreated with overlapping ablations.

As (neo-)adjuvant chemotherapy is not standard of care conformal to national guidelines, no patients received neo-adjuvant or adjuvant systemic therapy [31]. However, patients did receive induction chemotherapy when initially unsuitable for resection/thermal ablation (downstaging chemotherapy), when chemotherapy was likely to reduce procedural risk (risk-reducing chemotherapy) or when liver metastatic disease developed early after primary tumor diagnosis (test-of-tumor biology). MSI, RAS-, and BRAF-mutation status were not routinely established nor taken into account when discussing local treatment options for patients in the multidisciplinary tumor board.

### 2.3. Follow-up

In the first year 3/4-monthly, in the 2nd and 3rd year 6-monthly, and in the 4th and 5th year 12 monthly after local treatment ^18^F-FDG-PET-CT with diagnostic ceCTs of the chest and abdomen were performed, according to national guidelines [31]. A ceMRI with diffusion-weighted images was used as problem solver. A ceCT-scan was performed within one to six weeks after the repeat local treatment, in the context of a presumably incomplete local treatment procedure. LTP was defined as an unequivocally and solid enlarging mass or focal ^18^F-FDG PET avidity at the surface of the ablated tumor for thermally ablated tumors or histopathological confirmation in case of uncertainty.

### 2.4. Data Collection and Statistical Analysis

Patient, procedure, and tumor characteristics were obtained from the AmCORE database. Categorical variables are reported as percentages and continuous variables are reported as mean with standard deviation (SD) when normally distributed and as median with interquartile range (IQR) when not-normally distributed.

The assessed endpoints were local tumor progression-free survival (LTPFS), local control (LC), distant progression-free survival (DPFS), and overall survival (OS), defined as time-to-event from local treatment. Death without local or distant progression (competing risk) was censored. Complications were reported using Common Terminology Criteria for Adverse Events (CTCAE) [32]. Outcomes following local treatment were analyzed for primary tumor sidedness of colorectal cancer (CRC), RAS, and BRAF mutations and MSI status. Kaplan–Meier curves and Gehan–Breslow–Wilcoxon test was used to estimate and compare the primary endpoints LTPFS, to account for the events at early time points as LTP develops mostly early after local treatment. Kaplan–Meier method curves and the log-rank test was used to estimate and compare OS, DPFS, and LC. The Pearson chi-square test was used to compare complications. Uni- and multivariable analyses of primary endpoint LTPFS was performed based on Cox proportional hazards regression comparing right-sided CC, to left-sided CC, and to RC. Variables with *p* ≤ 0.050 in univariable analysis were included in multivariable analysis using backward selection procedure. Variables were considered significant when *p* = 0.050, other variables were removed step by step. Hazard ratio (HR) and 95% confidence interval (95% CI) were evaluated.

Statistical analyses were supported by an biostatistician (BLW) and performed using SPSS^®^ Version 24.0 (IBM^®^ Corp, Armonk, NY, USA) [33] and R version 4.0.3. (R Foundation, Vienna, Austria) [34].

## 3. Results

**Figure 1:**
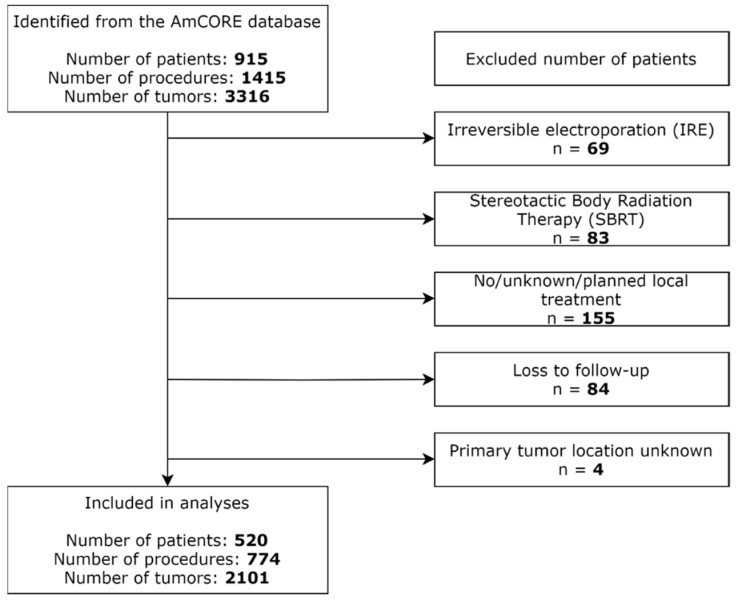
Flowchart of included and excluded patients.

A total of 915 patients, 1415 procedures, and 3316 tumors were identified from the AmCORE database (Figure 1). Further selection revealed 520 patients, 774 procedures, and 2101 tumors undergoing local treatment between 2000 and 2021 for inclusion in the analyses. Patients treated with IRE (*n* = 69), SBRT (*n* = 83), no, unknown, or planned local treatment (*n* = 155), with loss to follow-up (*n* = 84) and with unknown primary tumor location (*n* = 4) were excluded.

### 3.1. Patient-, Disease-, Procedure-, and Tumor-Related Characteristics

Table 1 presents baseline patient, procedure and tumor characteristics of the included 520 patients, 774 procedures, and 2101 tumors. The age of the treated patients ranged between 22 and 90 years old. Overall, median number of tumors per procedure was 2.0 (IQR 1.0–4.0) and median size per tumor was 16 mm (IQR 9.0–25.0). Median follow-up time after local treatment was 19.5 months and median length of hospital stay of the entire cohort was 4.0 days (IQR 1.0–6.0).

### 3.2. Primary Tumor Sidedness

No differences in complication rates (*p* = 0.352) and grades (*p* = 0.485) were found following local treatment of CRLM originating from right-sided CC, left-sided CC, and RC with complications in 24.2% in the right-sided CC group, 26.7% in the left-sided CC group, and 30.5% in the RC group (overall complication rate of 27.2%) (Table 2).

**Figure 2:**
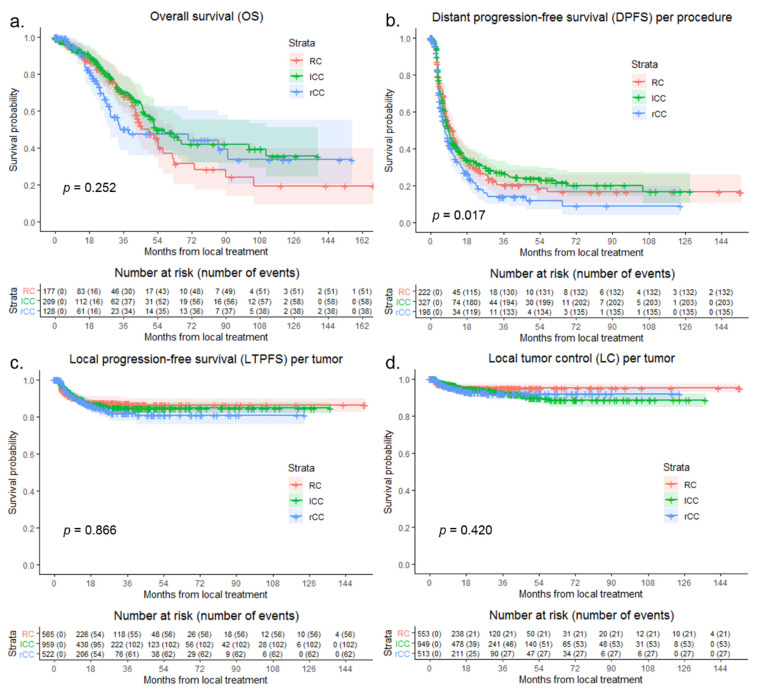
Kaplan–Meier curves, with log-rank (Mantel–Cox) test or Gehan–Breslow–Wilcoxon test, of (**a**) overall survival (OS) per patient (*p* = 0.252), (**b**) distant progression-free survival (DPFS) per procedure (*p* = 0.017), (**c**) local tumor progression-free survival (LTPFS) per tumor (*p* = 0.866), and (**d**) of local control (LC) per tumor (*p* = 0.420) after local treatment comparing RC (pink), left-sided CC (green) and right-sided CC (blue). Numbers at risk (number of events) are per patient, per procedure and per tumor. Death without distant progression, local tumor progression (LTP) or loss of LC (competing risk) is censored.

Median OS of the entire cohort was 52.0 months. Median OS following local treatment in the right-sided CC group was 38.8 months, in the left-sided CC group 52.9 months, and in the RC group 45.5 months (Figure 2a). In the right-sided CC group, 1-year OS was 90.7%, 3-year OS was 50.2%, and 5-year OS was 47.5%. In the left-sided CC group, 1-, 3-, and 5-year OS were, respectively, 92.9%, 69.9%, and 48.2%. Then, for 1-, 3-, and 5-year OS, respectively, 92.1%, 69.5%, and 37.1% were found in the RC group. Compared to right-sided CC (reference HR 1.000), OS was not significantly different for left-sided CC and RC (*p* = 0.254) with an HR for left-sided CC of 0.725 (95% CI, 0.481–1.093) and for RC of 0.917 (95% CI, 0.601–1.398).

Median DPFS following local treatment in the right-sided CC group was 6.9 months, in the left-sided CC group 9.0 and in the RC group 10.5 months (Figure 2b). In the right-sided CC group, 1-year DPFS was 35.3%, 3-year DPFS was 14.1% and 5-year DPFS was 12.1%. In the left-sided CC group, 1-, 3-, and 5-year DPFS were, respectively, 42.2%, 27.2%, and 23.2%. Then, for 1-, 3-, and 5-year DPFS, respectively, 44.8%, 20.3%, and 16.7% were found in the RC group. Compared to right-sided CC (reference HR 1.000), DPFS was superior for left-sided CC and RC (*p* = 0.018) with an HR for left-sided CC of 0.742 (95% CI, 0.596–0.923) and for RC of 0.760 (95% CI, 0.597–0.966).

LTP was reported at follow-up after local treatment of CRLM in 10.8% of tumors, 11.9% in right-sided CC group, 10.6% in the left-sided CC group, and 9.9% in the RC group (Figure 2c). Then, for 1-, 3-, and 5-year LTPFS, respectively, 89.0%, 81.9% and 80.7% were found in the right-sided CC group; 89.4%, 84.7%, and 84.7% in the left-sided CC group; and 89.2%, 87.1%, and 86.2% in the RC group.

No significant difference in crude, univariable comparison of LTPFS following local treatment was found (*p* = 0.559) between right-sided CC and left-sided CC (HR, 0.873; 95% CI, 0.636–1.197) and between right-sided CC and RC (HR, 0.843; 95% CI, 0.587–1.210). Factors potentially associated with LTPFS found in univariable analysis, as well as not significant associated variables in multivariable analysis are demonstrated in Table 3. Corrected multivariable analysis consisted of number of tumors at first diagnosis (*p* = 0.002), approach (*p* < 0.001), anesthesia technique (*p* < 0.001), appearance of CRLM (new vs. local recurrence, *p* < 0.001), size of metastasis (*p* = 0.006), and margin size (*p* < 0.001). Adjusted HR of left-sided CC compared to right-sided CC was 1.000 (95% CI, 0.654–1.530) and RC compared to right-sided CC was 0.931 (95% CI, 0.594–1.460) (*p* = 0.939).

Eventual loss of LC at follow-up was reported in 5.0% of tumors, 5.3% in the right-sided CC group, 5.6% in the left-sided CC group, and 3.8% in the RC group after local treatment (Figure 2d). Then, for 1-, 3-, and 5-year LC, respectively, 95.0%, 91.8%, and 91.8% were found in the right-sided CC group; 96.5%, 92.4%, and 88.4% in the left-sided CC group; and 95.7%, 95.1%, and 95.1% in the RC group. Compared to right-sided CC (reference HR 1.000), LC was not significantly different for left-sided CC and RC (*p* = 0.423) with an HR for left-sided CC of 0.956 (95% CI, 0.601–1.521) and for RC of 0.706 (95% CI, 0.399–1.249).

### 3.3. RAS and BRAF Mutations and MSI Status

With a complication rate of 24.6% in the RAS mutation group and 25.0% in the RAS-wildtype group (*p* = 0.961), the frequencies (*p* = 0.961) did not differ, nor did the grades (*p* = 0.964). No significant differences in complication rates were found for BRAF (*p* = 0.586) and MSI (*p* = 0.346) either (Table 2).

**Figure 3:**
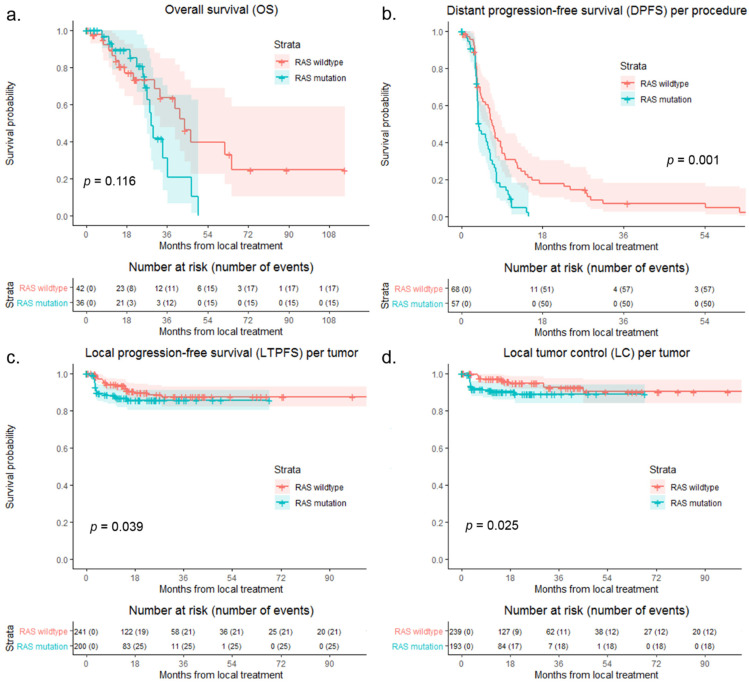
Kaplan–Meier curves, with log-rank (Mantel–Cox) test or Gehan–Breslow–Wilcoxon test, of (**a**) overall survival (OS) per patient (*p* = 0.116), (**b**) distant progression-free survival (DPFS) per procedure (*p* = 0.001), (**c**) local tumor progression-free survival (LTPFS) per tumor (*p* = 0.039), and (**d**) of local control (LC) per tumor (*p* = 0.025) after local treatment comparing RAS wildtype (blue) to RAS mutation (pink). Numbers at risk (number of events) are per patient, per procedure and per tumor. Death without distant progression, local tumor progression (LTP), or loss of LC (competing risk) is censored.

A total of 43 patients with RAS wildtype and 36 patients with RAS mutation were identified from the AmCORE database. Comparing RAS wildtype to RAS mutation, 86.9% of tumors had RAS mutation in the right-sided CC group, 27.9% in the left-sided CC group, and 40.7% in the RC group (*p* < 0.001). Figure 3 shows survival curves of OS, DPFS, LTPFS, and LC comparing RAS wildtype to RAS mutation. No significant difference was found in OS (*p* = 0.116). DPFS (*p* = 0.001), LTPFS (*p* = 0.039), and LC (*p* = 0.025) were significantly worse in the RAS mutation group.

**Figure 4:**
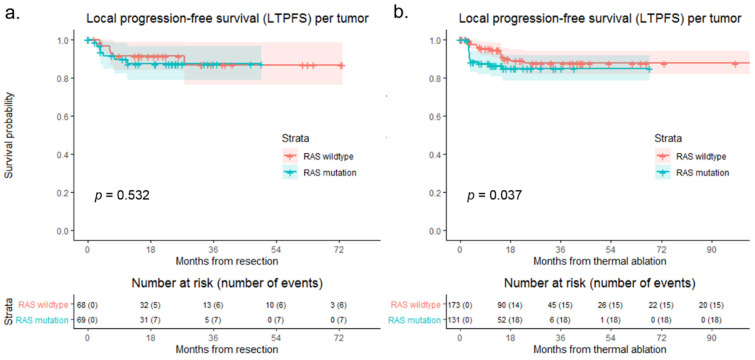
Kaplan–Meier curves with Gehan–Breslow–Wilcoxon test, of (**a**) local tumor progression-free survival (LTPFS) per tumor following resection (*p* = 0.532) and (**b**) LTPFS per tumor following thermal ablation (*p* = 0.037) comparing RAS wildtype (blue) to RAS mutation (pink). Numbers at risk (number of events) are per tumor. Death without local tumor progression (LTP; competing risk) is censored.

Subgroup analyses of LTPFS for resection and thermal ablation comparing RAS wildtype to RAS mutation are shown in Figure 4. No difference was found in LTPFS comparing RAS wildtype to RAS mutation following resection (*p* = 0.532). LTPFS was significantly lower in the RAS mutation group compared to the RAS wildtype group following thermal ablation (*p* = 0.037).

Identification of patients from the AmCORE database with BRAF wildtype/mutation revealed 63 patients with BRAF wildtype and 6 patients with BRAF mutation. Comparing BRAF wildtype to BRAF mutation, 11.6% of tumors had BRAF mutation in the right-sided CC group, 4.7% in the left-sided CC group, and 0.0% in the RC group (*p* = 0.001). OS (*p* < 0.001) was significantly worse in the BRAF mutation group. DPFS (*p* = 0.075), LTPFS (*p* = 0.679), and LC (*p* = 0.968) were not significantly different comparing BRAF wildtype to BRAF mutation.

A total of 124 patients with MSS and 4 patients with MSI were revealed from the AmCORE database. Comparing MSS to MSI, 3.5% of tumors had MSI in the right-sided CC group, 3.0% in the left-sided CC group, and 0.0% in the RC group (*p* = 0.073). OS (*p* < 0.001) was significantly lower in the MS instability group. DPFS (*p* = 0.316), LTPFS (*p* = 0.342), and LC (*p* = 0.968) were not significantly different comparing MSS to MSI.

## 4. Discussion

Compared to right-sided CC, DPFS was superior for left-sided CC and RC (*p* = 0.018) with an HR for left-sided CC of 0.742 (95% CI, 0.596–0.923) and for RC of 0.760 (95% CI, 0.597–0.966). DPFS (*p* = 0.001), LTPFS (*p* = 0.039), and LC (*p* = 0.025) were significantly lower for patients with RAS mutated tumors. Though, in subgroup analysis, no difference in LTPFS was found between RAS wildtype and RAS mutated CRLM following resection (*p* = 0.532), LTPFS was worse for RAS mutated tumors compared to RAS wildtype tumors following thermal ablation (*p* = 0.037). OS was significantly lower in the BRAF mutation group (*p* < 0.001), while BRAF mutation did not significantly affect DPFS (*p* = 0.075), LTPFS (*p* = 0.679) and LC (*p* = 0.968). In the MSI group, OS (*p* < 0.001) was significantly lower.

These results imply that information regarding the sidedness of the primary tumor, RAS and BRAF mutations and MSI status are prognostic biomarkers that preferably should be taken into consideration when discussing curative intent therapeutic options for locally treatable CRLM patients. Given the higher LTP rate, physicians should endeavor wider safety-margins for resections and thermal ablation for RAS mutated CRLM.

Compared to left-sided CRC, Dupré et al. showed decreased OS rates (34.6 versus 45.3 months, *p* = 0.035) [24] and Creasy et al. demonstrated an inferior HR of 1.22 (95% CI, 1.02–1.45; *p* = 0.028) for resected CRLM from right-sided CC [25]. Similar to our series, Makowiec et al. showed no significant difference in 5-year OS between patients with resected CRLM originating from right- versus left-sided CRC (*p* = 0.64) [23]. Gu et al. [27] found an inferior OS (*p* = 0.042) and higher recurrence rates (*p* = 0.029) for right-sided versus left-sided CRC following RFA. Though Zhou et al. did find a difference in progression-free survival favoring left-sided CRC (*p* = 0.012), there was no significant difference in OS (*p* = 0.583) following MWA [28].

RAS mutations are present in approximately 40% of patients with CRC, substantially affecting survival outcomes following primary tumor resection and CRLM resection [35,36,37,38]. Zhang et al. revealed increased rates of micrometastases (*p* < 0.001) and reduced margin size resulting in increased R1 resections (*p* = 0.002) in patients with KRAS mutations following liver surgery [39]. In multivariate analysis, KRAS mutation (HR, 1.495; 95% CI, 1.069–2.092; *p* = 0.019) and margin status (HR, 1.560; 95% CI, 1.017–3.033; *p* = 0.043) were significantly correlated with hepatic disease recurrence. Therefore, in patients with KRAS mutated CRLMs, Zhang et al. advocated wider surgical margins. In addition, Brudvik et al. reported an association between RAS mutation and positive margins in multivariate analysis (HR, 2.439; 95% CI, 1.300–4.575; *p* = 0.005) [40]. RAS mutation (HR, 1.629; 95% CI, 1.013–2.620; *p* = 0.044) and positive margins (HR, 3.360; 95% CI, 1.741–6.485; *p* < 0.001) both correlated with reduced OS rates in multivariate analysis. Brudvik et al. proposed margin sizes over 10 mm, as suggested earlier by Are et al. as well [40,41]. Achterberg et al. showed that near-infrared fluorescence imaging using indocyanine green may contribute to acquiring these tumor-negative resections in minimal invasive surgery [42].

To assess the prognostic role of RAS status, Odisio et al. compared patients with RAS wildtype to patients with RAS mutation following thermal ablation [43]. LTP was found in 39% of patients with RAS mutated CRLMs and in 14% of patients with RAS wildtype CRLMs (*p* = 0.007). Three-year LTPFS was 35% compared to 71% of, respectively, RAS mutation and RAS wildtype (HR 3.01, 95% CI 1.60–5.77, *p* = 0.001). Furthermore, Shady et al. showed KRAS to be a significant predictor of LTPFS following thermal ablation in univariate analysis (HR, 1.5; 95% CI, 0.89–2.7; *p* = 0.12) [44]. They proposed peri-ablational safety margins >6 mm to obtain LC in patients with KRAS mutated CRLMs, as margins of 1–5 mm after thermal ablation in the KRAS group showed inferior LTP rates (*p* = 0.018) [44]. Similar to our work, Odisio et al. and Shady et al. performed ablations without consideration of RAS mutation status. Based on these and other previous studies and based on the currently presented results, we suggest peri-ablational minimum safety margins of >5 mm for RAS-wildtype and >10 mm for RAS-mutated CRLM [40,43,45,46,47,48,49]. Recent and ongoing developments in thermal ablation techniques, comprising antenna, probe, and generator design, improved image-guidance, real-time navigation, and image-fusion and registration and the use of confirmation software to reliably assess margins, contribute in the achievement of these sufficient peri-ablational safety margins [45,46,47,48,49,50,51].

The high number of patients, procedures and tumors allowed sufficiently powered statistical analyses, which strengthened this study. The choice of treatment and patient selection was based on multidisciplinary tumor board evaluations where primary tumor sidedness, RAS and BRAF mutations, and MSI status were previously disregarded in decision making. Though confounding was limited by the use of Cox proportional hazards and regression models including multivariable analysis, residual confounding cannot be ruled out regarding the subgroup analysis for resection and thermal ablation. Because RAS, BRAF, and MSI status were not routinely established and, hence, often unknown, the analyses of the impact of RAS and BRAF mutations and MSI status were less robustly powered, increasing the risk of bias. Additionally, technical developments in resection and thermal ablation over the long study period may have caused population bias and the local treatment techniques do not automatically represent the current global standards of care.

## 5. Conclusions

To conclude, for patients undergoing partial hepatectomy or thermal ablation for CRLM, BRAF mutations, and, if not treated with immunotherapy, MS instability were associated with a higher probability of relapse and death. RAS mutated tumors were at higher risk for an incomplete resection or ablation, suggesting the necessity to widen margins whenever feasible. Margins >5 mm and >10 mm are advised for RAS wildtype and RAS mutated CRLM, respectively. Knowledge regarding BRAF and RAS mutations and MSI status improves clinical judgment and decision making and it may improve outcome by reducing the number of repeat treatments.

## Figures and Tables

**Table 1:** Patient-, procedure-, and tumor-related characteristics.

Patient-Related Characteristics		*n* = 520
Gender	Male	66.5
	Female	33.5
Age (years) *		65.5 (12.0)
ASA physical status	1	7.0
	2	69.9
	3	22.7
	4	0.4
Comorbidities	None	49.2
	Minimal	37.0
	Major	13.8
BMI (kg/cm^2^) *		25.8 (4.4)
**Disease-Related Characteristics**		***n* = 520**
First diagnosis of CRLM	Synchronous	50.6
	Metachronous	49.4
Size of largest metastasis at first diagnosis of CRLM (mm)	Small (1–30)	63.6
	Intermediate (31–50)	24.5
	Large (>50)	11.9
Number of tumors at first diagnosis of CRLM	1	36.3
	2–5	48.1
	>5	15.6
Extrahepatic disease at first diagnosis of CRLM	No	89.4
	Yes	10.6
Primary tumor location	Right-sided CC	25.0
	Left-sided CC	40.4
	RC	34.6
RAS status (any)	RAS wildtype	8.3
	RAS mutation	6.9
	Unknown	84.8
BRAF V600 status	BRAF wildtype	12.1
	BRAF mutation	1.2
	Unknown	86.7
MSS/MSI status	MSS	23.8
	MSI	0.8
	Unknown	75.4
**Procedure-Related Characteristics**		***n* = 774**
Local treatment	Resection	27.3
	Thermal ablation	48.3
	Combination	24.4
Chemotherapy	No	76.1
	Yes	23.9
Procedure number in course of treatment	1st	67.4
	2nd–5th	32.6
Number of tumors	1	38.3
	2–5	48.7
	>5	13.1
Approach	Open	58.4
	Laparoscopic	7.8
	Percutaneous	33.8
Anesthesia	Midazolam sedation	6.1
	Propofol sedation	16.8
	General anesthesia	82.8
**Tumor-Related Characteristics**		***n* = 2101**
Size (mm)	Small (1–30)	84.5
	Intermediate (31–50)	12.4
	Large (>50)	3.1
Margin size (mm)	0–5	13.8
	>5	86.2
Local treatment	Resection	36.6
	Thermal ablation	63.4

Values are reported as percentage of patients, * = continuous variables reported as mean (standard deviation; SD), right-sided CC = right-sided colon cancer, left-sided CC = left-sided colon cancer, RC = rectal cancer, ASA = American Society of Anesthesiologists score, BMI = body mass index.

**Table 2:** Complications of local treatment per primary tumor location and RAS status (CTCAE) [32].

Grade	Total *n* = 774	Right-Sided CC *n* = 204	Left-Sided CC *n* = 336	RC *n* = 234	*p*-Value	RAS-wt *n* = 43	RAS-mut *n* = 36	*p*-Value
Total	27.2	24.2	26.7	30.5	0.352 ^a^	24.6	25.0	0.961 ^a^
Grade 1	5.0	5.7	4.4	5.1		4.6	5.4	
Grade 2	8.6	7.3	8.5	9.8		9.2	10.7	
Grade 3	10.1	7.3	9.7	13.1		7.7	7.1	
Grade 4	2.1	3.6	1.9	0.9		1.5	1.8	
Grade 5	1.0	0.5	1.3	0.9	0.485 ^a^	1.5	0.0	0.964 ^a^

Values are reported as percentage of patients, right-sided CC = right-sided colon cancer, left-sided CC = left-sided colon cancer, RC = rectal cancer, RAS-wt = RAS wildtype, RAS-mut = RAS mutation, ^a^ = Pearson chi-square.

**Table 3:** Univariable and multivariable Cox regression analysis to detect variables associated with local tumor progression-free survival (LTPFS).

		Univariable Analysis		Multivariable Analysis	
		HR (CI)	*p*-Value	HR (CI)	*p*-Value
CRC	Right-sided CC	Reference	0.599	Reference	0.939
	Left-sided CC	0.873 (0.636–1.197)		1.000 (0.654–1.530)	
	RC	0.843 (0.587–1.210)		0.931 (0.594–1.460)	
**Patient-related factors**					
Gender	Male	Reference	0.494		
	Female	0.904 (0.676–1.208)			
Age (years)		1.007 (0.995–1.018)	0.249		
ASA physical status	1	Reference	0.224		
	2	1.060 (0.635–1.771)			
	3	0.722 (0.401–1.302)			
	4	*			
Comorbidities	None	Reference	0.824		
	Minimal	0.978 (0.736–1.300)			
	Major	0.871 (0.565–1.344)			
BMI (kg/cm^2^)		1.000 (0.971–1.029)	0.984		
First diagnosis of CRLM	Metachronous	Reference	<0.001	Reference	0.373
	Synchronous	0.560 (0.427–0.734)		1.191 (0.810–1.751)	
Size of largest metastasis at first diagnosis of CRLM (mm)	Small (1–30)	Reference	0.001	Reference	0.712
	Intermediate (31–50)	1.613 (1.176–2.213)		1.108 (0.704–1.742)	
	Large (>50)	1.903 (1.251–2.895)		1.336 (0.655–2.724)	
Number of tumors at first diagnosis of CRLM	1	Reference	<0.001	Reference	0.002
	2–5	0.617 (0.448–0.849)		0.931 (0.618–1.402)	
	>5	0.262 (0.176–0.391)		0.416 (0.242–0.714)	
Extrahepatic disease	No	Reference	0.807		
	Yes	0.941 (0.580–1.529)			
**Procedure-related factors**					
Local treatment	Resection	Reference	<0.001	Reference	0.055
	Thermal ablation	1.455 (1.000–2.118)		2.234 (1.157–4.312)	
	Combination	0.714 (0.471–1.081)		1.553 (0.806–2.991)	
Chemotherapy	No	Reference	<0.001	Reference	0.199
	Yes	0.485 (0.357–0.659)		0.753 (0.488–1.161)	
Procedure number in course of treatment	1st	Reference	<0.001	Reference	0.099
	2nd–5th	2.341 (1.785–3.069)		0.624 (0.356–1.093)	
Number of tumors	1	Reference	<0.001	Reference	0.385
	2–5	0.464 (0.339–0.633)		0.818 (0.492–1.360)	
	>5	0.160 (0.105–0.242)		0.542 (0.226–1.295)	
Approach	Open	Reference	<0.001	Reference	<0.001
	Laparoscopic	0.771 (0.315–1.885)		0.217 (0.030–1.567)	
	Percutaneous	2.999 (2.285–3.938)		3.138 (1.885–5.226)	
Anesthesia	Midazolam sedation	Reference	<0.001	Reference	<0.001
	Propofol sedation	0.192 (0.110–0.336)		0.260 (0.142–0.476)	
	General anesthesia	0.183 (0.124–0.270)		0.645 (0.344–1.208)	
**Tumor-related factors**					
Appearance	New tumor	Reference	<0.001	Reference	<0.001
	Local recurrence	5.527 (4.119–7.417)		2.675 (1.742–4.107)	
Size of metastasis (mm)	Small (1–30)	Reference	<0.001	Reference	0.006
	Intermediate (31–50)	2.229 (1.597–3.110)		1.575 (1.020–2.430)	
	Large (>50)	4.131 (2.465–6.923)		2.927 (1.392–6.157)	
Margin size	<5 mm	Reference	<0.001	Reference	<0.001
	>5 mm	0.382 (0.266–0.550)		0.424 (0.279–0.645)	

HR = hazard ratio, CI = 95% confidence interval, right-sided CC = right-sided colon cancer, left-sided CC = left-sided colon cancer, RC = rectal cancer, ASA = American Society of Anesthesiologists score, * insufficient group comparison, BMI = body mass index. Using backward selection procedure, results of step by step removed variables were reported. Results are from last step of removal.

## Data Availability

The data presented in this study are available on request from the corresponding author.

## Abbreviations

^18^F-FDG	[18F]-fluoro-2-deoxy-d-glucose
AmCORE	Amsterdam Colorectal Liver Met Registry
BRAF	V-raf Murine Sarcoma Viral Oncogene Homolog B
CC	Colon Cancer
Ce	Contrast-enhancement
CEA	Carcinoembryonic Antigen
CI	Confidence Interval
CRC	Colorectal Cancer
CRLM	Colorectal Liver Metastases
CT	Computed Tomography
CTCAE	Common Terminology Criteria for Adverse Events
DPFS	Distant Progression-free Survival
HR	Hazard Ratio
IRE	Irreversible Electroporation
IQR	Interquartile range
LTP	Local Tumor Progression
LTPFS	Local Tumor Progression-free Survival
MRI	Magnetic Resonance Imaging
MSI	Microsatellite Instability
MWA	Microwave Ablation
OS	Overall Survival
PET	Positron Emission Tomography
RAS	Rat Sarcoma Viral Oncogene Homolog
RC	Rectal Cancer
RFA	Radiofrequency Ablation
RR	Risk Ratio
SBRT	Stereotactic Body Radiation Therapy
SD	Standard Deviation
STROBE	Strengthening the Reporting of Observational studies in Epidemiology
